# Photophysical and anion sensing properties of a triphenylamine–dioxaborinine trimeric compound†

**DOI:** 10.1039/d2ra07498b

**Published:** 2023-01-09

**Authors:** Alexis Tigreros, Camilo Bedoya-Malagón, Alejandra Valencia, Mayerlin Núñez-Portela, Jaime Portilla

**Affiliations:** a Bioorganic Compounds Research Group, Department of Chemistry, Universidad de Los Andes Carrera 1 No. 18A-10 Bogotá 111711 Colombia jportill@uniandes.edu.co; b Quantum Optics Laboratory, Department of Physics, Universidad de Los Andes Carrera 1 No. 18A-10 Bogotá Colombia

## Abstract

Herein, we report the synthesis and photophysical characterization of the novel tris(4-(2,2-difluoro-6-methyl-2*H*-1λ^3^,3,2λ^4^-dioxaborinin-4-yl)phenyl)amine trimeric probe (A2) *via* the reaction between triphenylamine (1), acetic anhydride, and BF_3_·OEt_2_ implying the twelve new bond formation in a one-pot manner. This highly fluorescent compound in solution (*φ* up to 0.91 at 572 nm) and solid state (*φ* = 0.24 at 571 nm) showed a better solvatofluorochromism than its analog monomeric A1 due to symmetry-broken charge transfer, which is consistent with high solvent dipolarity (SdP) response in Catalán's multiparametric regression. Notably, A2 had a high sensibility and selectivity for CN^−^ or F^−^ in solution (LODCN^−^/F^−^ = 0.18/0.70 μM), and CN^−^ can be discriminated from F^−^ by the reaction of A2 with 3.0 equiv. of CN^−^. In addition, A2 was impregnated on filter paper to prepare test strips that were applied to naked-eye qualitative sensing of CN^−^ or F^−^. Finally, the octupolar system in A2 allows for better action of two-photon excitation cross-section values when compared with that of the dipolar structure in A1. These findings provide further information for the design of new efficient two-photon absorption dyes.

## Abstract 2

The one-pot triphenylamine functionalization with multiple dioxaborinine groups gives the novel dye A2 with two-photon absorption and cyanide-sensing properties. The solvatofluorochromism of A2 was interpreted in terms of solvent dipolarity.

## Graphical abstract

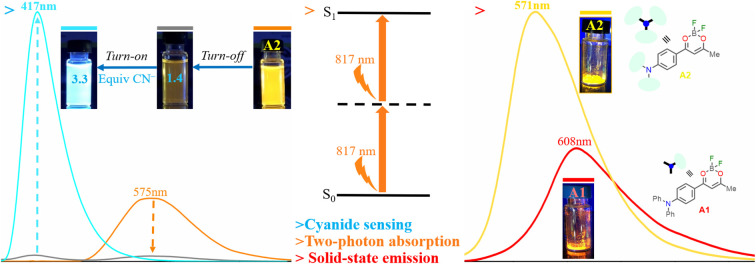

## Introduction

Boron complex-containing molecules are an interesting and extensively used class of organic fluorescent compounds. These dyes have valuable photophysical properties such as strong absorption bands, high fluorescent quantum yields (*φ*), good solubility in organic solvents, photostability, microenvironment-dependent emission, *etc.*^1–3^ Therefore, this important family of organic fluorophores can serve in numerous applications involving bioimaging probes,^4^ photosensitizers in photodynamic therapy,^5^ red-emitting complexes with a mega-large Stokes shift,^6^ multicolor fluorescent initiators,^7^ colored triboluminescence compounds,^8^ and fluorescent probes for mercury detection in living cells,^7^ among others.

Importantly, some characteristic fluorophores perform through two-photon absorption (TPA) phenomena that involve the simultaneous absorption of two photons from a laser light source,^9^ which has advantages over the classical one-photon process. For instance, a molecule can be excited with low-energy photons *versus* the classical methods. The TPA transition probability increases with the excitation laser intensity's square giving the optical absorption's high spatial selectivity,^10^ leading to applications in three-dimensional (3D) data storage,^11^ photodynamic therapy,^12,13^ 3D micro-fabrication,^14^ and high-resolution bioimaging.^15^ Thus, novel fluorophores development by efficient synthetic approach is highly desirable and a research-active field in chemistry, material sciences, and the industry.

Structurally, many architectures have been used to achieve good TPA responses; that is, conjugated donor (D) and acceptor (A) with different geometry organizations, dipolar (D–π–A), quadrupolar (D–π–A–π–D or A–π–D–π–A), and octupolar (D–(π–A)_3_ or A–(π–D)_3_),^16^ this appreciation has been verified by theoretical and experimentally results in the Jean-Luc Brédas research group.^17^ In this way, the electronic connecting between strategic fluorophores is a good tactic for designing dyes with TPA properties; for example, hybrid fluorophores containing triphenylamine (TPAm) or the boron complex dioxaborinine (DB) have been used for this purpose.^9,18^

**Scheme 1:**
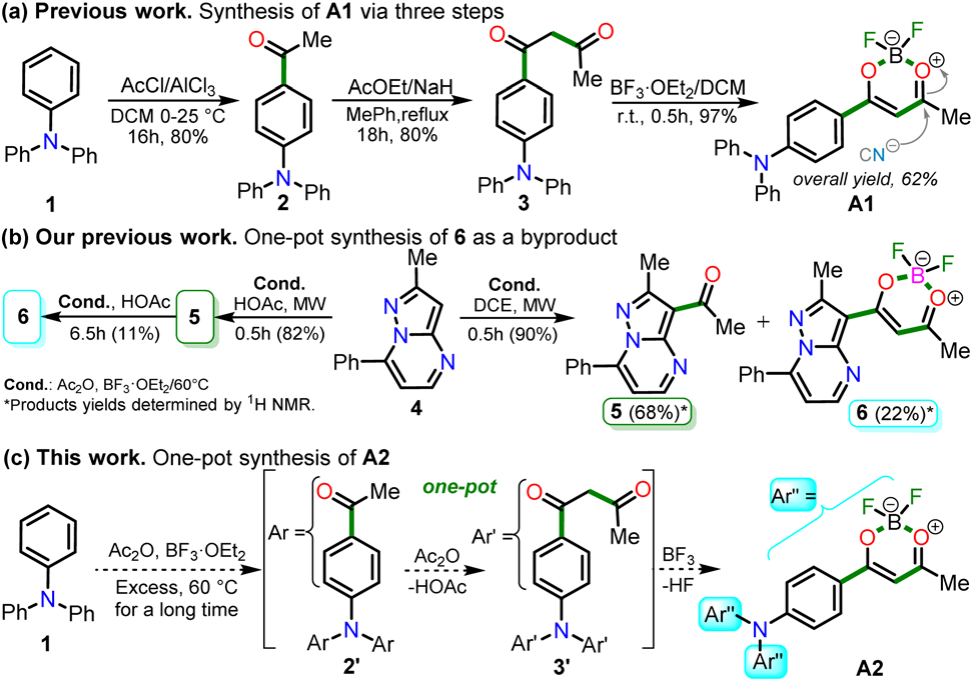
Synthesis of fluorophores bearing dioxaborinine (a) A1,^19^ (b) 5, and (c) A2.^20^

**Scheme 2:**
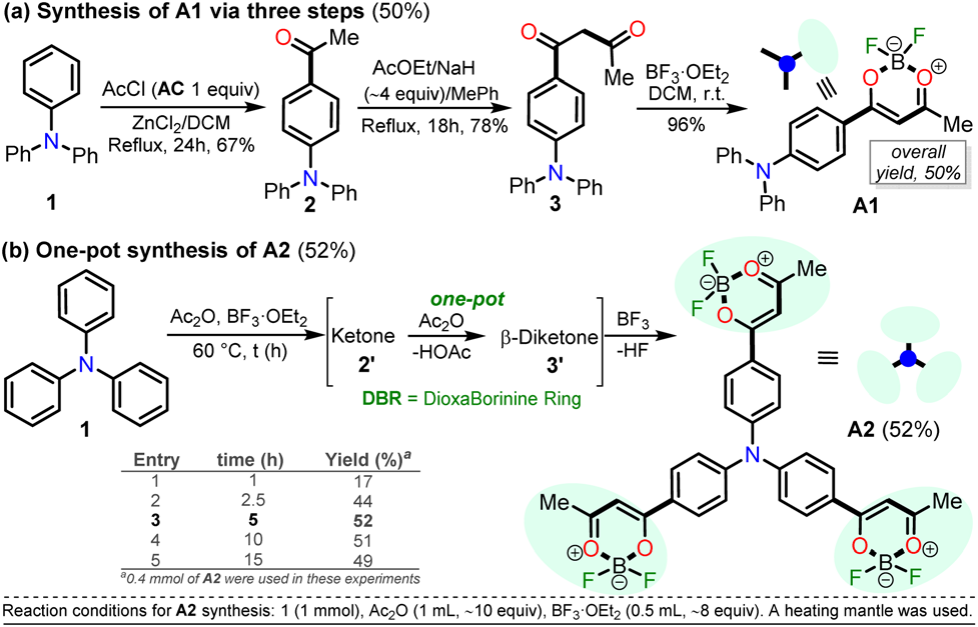
Synthesis of dioxaborinine–triphenylamine hybrid dyes (a) A1 and (b) A2.

In 2020, Tamilarasan *et al.*^19^ synthesized the dipolar dye A1 (Scheme 1a) based on a triphenylamine–dioxaborinine hybrid compound as a colorimetric and fluorimetric probe for the reversible detection of cyanide (LOD = 0.36 μM in MeCN : H_2_O 98 : 2). The probe was obtained in 62% global yield by a three-step sequence starting from TPAm (1). This synthesis involves a sequential double acetylation reaction *via* the carbonyl compounds intermediates 2 and 3, and the final formation of the boron complex in A1. Recently, we carried out a BF_3_-mediated synthesis of 3-acetylpyrazolo[1,5-*a*]pyrimidines (similar to 5) under microwave (MW) irradiation and using acetic anhydride as an acetylating agent (Scheme 2b). During the optimizing reaction conditions process, we observed that the fluorescent by-product 6, bearing the pyrazolo[1,5-*a*]pyrimidine–dioxaborinine hybrid system resulting from the formation of four new bonds in a one-pot manner, is favored in long reaction times.^20^

Within the most conventional photophysical applications of organic fluorophores, ion recognition has been the subject of extensive study in the last two decades;^21,22^ in particular, cyanide (CN^−^) is one of the most concerning environmental ions. Cyanide toxicity is known because it can inhibit the mitochondrial cytochrome C oxidase and suppress oxygen transport.^23,24^ Consequently, the development of sensitive and selective probes for the sensing of this anion has been gaining considerable attention in recent years because of its essential role in a wide variety of biological,^25^ environmental,^26^ biochemical,^27^ clinical,^28^ synthetical due to design and preparation of the respective chemosensors and industrial applications.^21,22^

Considering the above background and our continuing interest in obtaining new functional fluorophores,^20,29–31^ we planned to develop the octupolar dye A2 by a synthesis implying the twelve new bonds formation in a one-pot manner (Scheme 2c). Fluorophore A2 is a tris-dioxaborinine–triphenylamine hybrid compound with promising photophysical properties in solution and solid-state, which we wish to study and compare with those for the similar dipolar system A1. Explicitly, A2 is expected to have good TPA responses with improved results over other octupolar dyes synthesized even by difficult methods involving various reaction steps;^32^ for this purpose and as a proof-of-concept, by measuring the two-photon excitation action cross section when exciting the probes A1 and A2 with a continuous-wave (CW) laser at 810 nm. Ultimately, similar to A1,^19^ the novel dye A2 could be applied for cyanide sensing by fluorescent changes after a chemical reaction with the anion.

## Results and discussion

### Synthesis

For this research, the dioxaborinine–triphenylamine hybrid dyes A1 and A2 were synthesized. The synthetical approaches for preparing these dyes starting from triphenylamine (1, TPAm), are simple and proceed in good yields (Scheme 2). According to the literature,^19,33,34^ compound A1 was synthesized in 50% global yield *via* a three-step sequence starting from triphenylamine (1). Nevertheless, for A2 synthesis, a new approach was used to convert substrate 1 into the trisubstituted derivate *via* the twelve new bonds formation in a one-pot manner in good yield (52%). In this respect, the mixture of acetic anhydride (AA) with boron trifluoride diethyl etherate (BF_3_·OEt_2_) was used as multiple acetylating agents six times to give the respective β-diketone intermediate 3′ and the complexing agent of the last step. Structures for the hybrid dyes A1 and A2 were established by NMR spectroscopy (^1^H, ^13^C, and ^19^F) and HRMS analysis (Fig. S1–S9†).

Remarkably, although the hybrid compounds A1 (50%) and A2 (52%) were obtained with close yields starting from triphenylamine (1), the octupolar dye A2 requires only one step for its formation in 5 hours at 60 °C and with the twelve new bonds formation. In contrast, the dipolar derivative A1 synthesis (Scheme 2a) consumptions three steps (*i.e.*, ring acetylation/one new bond, ketone α-acetylation/one new bond, and complexation with BF_3_/two new bond) through the intermediates (4-acetylphenyl)diphenylamine (2) and 1-(4-(diphenylamino)phenyl)butane-1,3-dione (3). Thus, the synthesis of A2 implies a much better operational simplicity, lower consumption of solvents due to the solvents used as reaction medium and in the purification steps and a more excellent atomic economy concerning the probe A1 as a result of the one-pot synthesis of A2.

It is important to note that the reaction time played a crucial role in A2 synthesis since, after 1 hour at 60 °C, the product was obtained with only a 17% yield; however, the yield increased to 44% after 2.5 hours of reaction. The optimal conditions for this reaction turned out to be 5 hours at 60 °C because the yield increased to 52%, and no noticeable changes were observed during longer reaction times; with lower temperatures, the reaction does not progress much, and with higher temperatures, by TLC, a complex mixture of products was observed (Scheme 2b). Consequently, our previous results on constructing the dioxaborinine ring are a reliable starting point for accessing various derivatives of this heterocyclic core.^20^ Additionally, it was possible to establish that the optimum temperature to treat the acetylating mixture (an excess of Ac_2_O/BF_3_·OEt_2_) is 60 °C. Finally, we could verify our hypothesis that the formation of the DB ring in a one-pot manner is enhanced when long reaction times are used.

### Photophysical properties

#### Solvatofluorochromism

**Fig. 1:**
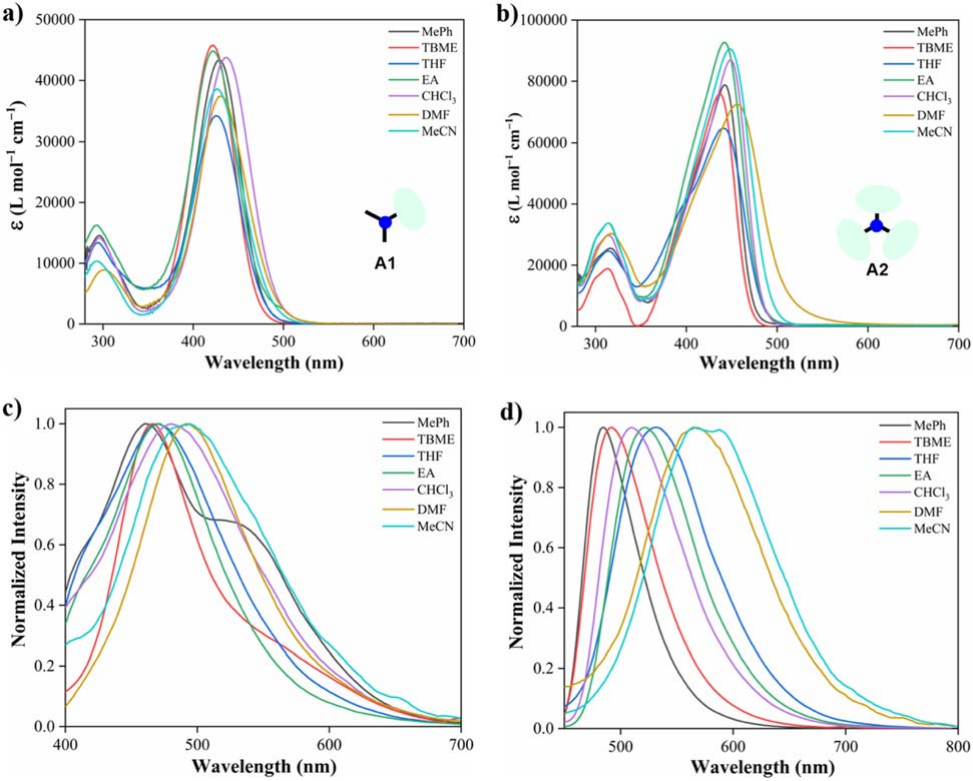
Absorption and emission (*λ*_ex_ = 350 nm) spectra in various solvents (10.0 μM) at 20 °C of hybrid compounds A1 (a and c) and A2 (b and d).

Solvent-dependent optical properties of compounds A1 and A2 were evaluated by UV-vis absorption and emission measurements in a set of non-protic solvents (Fig. 1, Table 1, and eqn (1); see the Experimental section) such as toluene (PhMe), *tert*-butyl methyl ether (TBME), tetrahydrofuran (THF), ethyl acetate (EA), chloroform (CHCl_3_), *N*,*N*-dimethylformamide (DMF), and acetonitrile (MeCN). For instance, in toluene, two absorption bands were observed for A1 (295 and 427 nm) and A2 (315 and 446 nm), the latter having a molar absorptivity at least twice that of A1. This feature is ascribed to the degeneracy of the S1 state of A2 and the increased number of DB groups that alter the electronic energy levels of the triphenylamine core by π-conjugation.^35^ Meanwhile, little to non-interaction at the ground state was noticed since there was no solvatochromism in A1 and A2.

**Table 1:** Photophysical properties of the hybrid compounds A1 and A2^a^

Entry	Solvent	A1		A2	
		*λ*_abs_ (nm), *ε* (L mol^−1^ cm^−1^)	*λ*_em_ (nm), *ϕ* (%)	*λ*_abs_ (nm), *ε* (L mol^−1^ cm^−1^)	*λ*_em_ (nm), *ϕ* (%)
1	PhMe	429, 43 300	461, 68	443, 78 700	484, 91
2	TBME	421, 45 700	467, 25	437, 76 000	492, 90
3	THF	425, 34 200	470, 12	440, 64 800	510, 29
4	EA	422, 44 800	470, 40	441, 92 600	522, 74
5	CHCl_3_	436, 43 800	480, 53	449, 87 100	531, 85
6	DMF	430, 37 300	492, 22	456, 72 400	567, 05
7	MeCN	426, 38 600	492, 13	457, 90 300	572, 08
8^b^	—	—	608, 11	—	571, 24

a Quantum yields (*ϕ*) were determined using Prodan as a reference standard (see eqn (1)).

b Solid-state.

Moreover, emission bands of A1 shift bathochromically from the weakly polar toluene (*λ*_em_ = 461 nm) to the strongly polar acetonitrile (*λ*_em_ = 492 nm); while A2 displays an intense solvatofluorochromism (*λ*_em_/MePh = 484 nm to *λ*_em_/MeCN = 575 nm), exhibiting a more dipolarity in the excited state, which may correspond to a symmetry broken dipolar state.^36^ Catalán multiparametric regression analysis was performed to evaluate the polarity at the excited state of A1 and A2 (eqn (2), Table S1†). The solvent dipolarity (SdP) stabilized best the excited state of A1 (slope = −1937.90 cm^−1^, *R*^2^ = 0.8862) and A2 (slope = −4607.54 cm^−1^, *R*^2^ = 0.9438). In general, compound A2 displays a more polar structure at the excited state when compared with A1 (Fig. S12 and S13†). This result is consistent with previous studies demonstrating the solvent-induced symmetry-breaking charge transfer in an octupolar chromophore.^35^ It is important to note that the branching increases the fluorescence quantum yield in a low to medium-polarity solvent (Table 1).

#### Solid-state emission

**Fig. 2:**
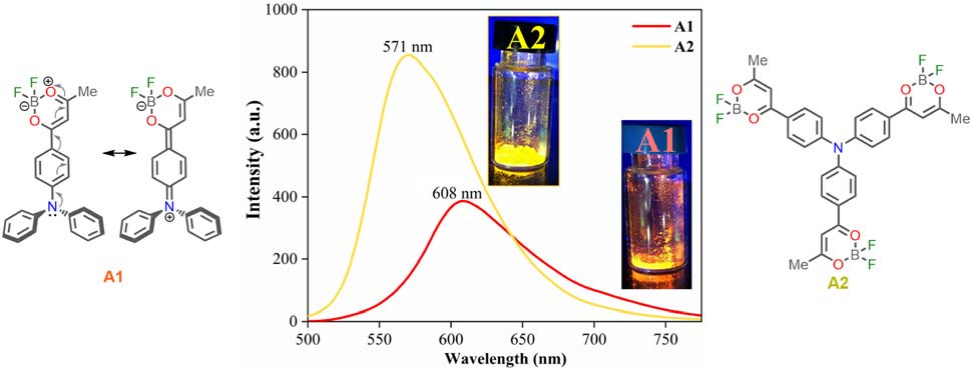
Fluorescence spectra for dyes A1 and A2 in the solid-state at 20 °C, *λ*_ex_ = 350 nm.

The fluorescence spectra in the solid-state of compounds A1 and A2 were registered under excitation at 300 nm at room temperature, and the results were fortunately very satisfactory (Fig. 2 and Table 1, entry 8). Emission of A1 in the solid-state falls in the orange-red region (608 nm) and that of A2 in the yellow-orange region (571 nm). Curiously, the solid-state emission spectrum of A2 resembles that of A2 dissolved in a high-polarity solvent. In contrast, the solid-state emission spectrum of the dipolar chromophore A1 is highly red-shift from that in solution (498 nm in MeCN). Such a marked difference in solid-state emission of the dyes A1 and A2 can be related possibly to the different packing of the dipolar and octupolar molecules in the solid state.^37^ Consequently, a strong intermolecular donor–acceptor interaction is expected in the solid phase for the dipolar dye A1. Another explanation could rely on the formation of J-aggregates that usually show different optical properties from dyes in solution, including red-shifted absorption and emission spectra and enhanced fluorescence quantum yields.^38^

#### Response to anions sensing in solution

**Fig. 3:**
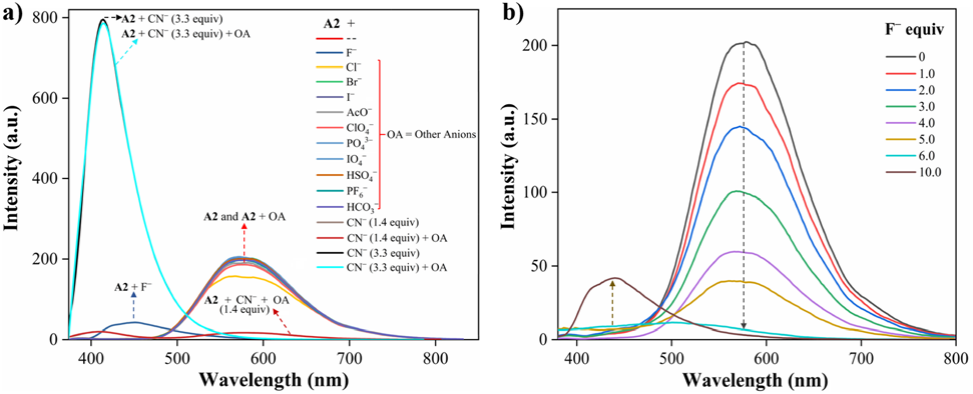
Emission spectra of A2 (10.0 μM, *λ*_ex_ = 350 nm) in MeCN/Tris (9 : 1) at 20 °C (a) with various anions (100.0 μM) and CN^−^ (14.0 and 33.0 μM), and (b) with F^−^ (0–100.0 μM).

Recently, Tamilarasan *et al.*^19^ studied compound A1 as a probe for cyanide sensing applications due to the presence of the dioxaborinine unit as an excellent cyanide-acceptor group. Inspired by these previous studies and our interest in developing molecular probes to detect cyanide,^21,22,31^ we envisioned dye A2 could show an interesting behavior in this sensing field. Thus, the anion sensing property of A2 was evaluated by treating the probe with CN^−^ and other anions of the potassium or sodium salts (100 μM), including F^−^, Cl^−^, Br^−^, I^−^, AcO^−^, IO_4_^−^, PO_4_^3−^, HSO_4_^−^, HCO_3_^−^, and ClO^−^ using MeCN/Tris (9 : 1, 1.0 mM at pH 7.5) as a solvent. When CN^−^ (1.4 equiv.) was added to the dye solution, the emission band around 572 nm disappeared, and the color of the solution changed from orange to pale yellow; in contrast, by adding other different anions, except by fluorine (F^−^), no substantial changes in the emission spectra were observed (Fig. 3a).

**Fig. 4:**
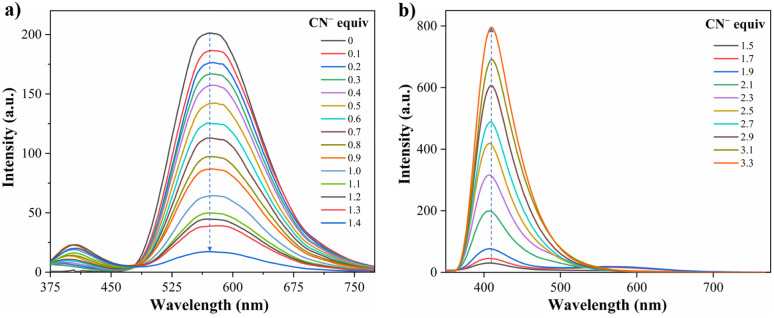
Emission spectra of A2 (10.0 μM, *λ*_ex_ = 350 nm) in MeCN/Tris (9 : 1) at 20 °C in the presence of CN^−^ (a) 0–14.0 μM and (b) 15.0–33.0 μM.

Due to the good preliminary results using the hybrid compound A2 for anions recognition, titration of A2 with F^−^ in acetonitrile/Tris was carried out the interaction was monitored by fluorescence at 572 nm (Fig. 3b). Upon the addition of 6.0 equiv. of F^−^, the emission band at 572 nm decreased linearly with a concentration (conc.) increased from of 0 to 60.0 μM; the limit of detection (LOD), calculated by the expression LOD = 3 × SD/*σ*, where *σ* is the slope of the titration curve, and SD is the standard deviation of ten measurements of the blank, for F^−^ was found to be 0.70 μM. These preliminary results allow us to conclude that the sensitivity of A2 toward CN^−^ is much higher than that found for F^−^; indeed, high fluorine concentrations are needed to complete the decrease in the emission band. Thus, titration with CN^−^ was also carried out to establish dye A2 as an efficient probe to detect cyanide (Fig. 4). The fluorescence intensity of the emission band at 572 nm in A2 decreased linearly when the CN^−^ concentration in the range of 0 to 14.0 μM (Fig. 4a). Noticeable, after 1.4 equiv. of CN^−^, a new emission band appears around 410 nm, and the fluorescence intensity of the band increased linearly with the concentration of CN^−^ in the range of 15.0 to 33.0 μM (Fig. 4b). Finally, the LOD for CN^−^ was evaluated to be 0.18 μM from Fig. S3 and S6.† These results indicate that A2 is a sensitive probe for CN^−^ sensing by fluorimetric methods, showing a LOD far below the WHO suggestion (1.9 μM) for drinking water.^39^ Moreover, comparing these results with those reported for A1 (LOD = 0.36 μM)^19^ indicates that an octupolar architecture improves the cyanide sensing performance.

Several chemosensors containing dioxaborinine core as the signaling subunit have been described recently; therefore, a representative summary of this type of probe compared to the synthesized dye in this work was carried out (Table 2).^19,40–42^

**Table 2:** Comparison of representative cyanide chemosensors, based on dioxaborinine core, with the new probe A2^a^

Compound	Features, LOD, and solvent	Reference
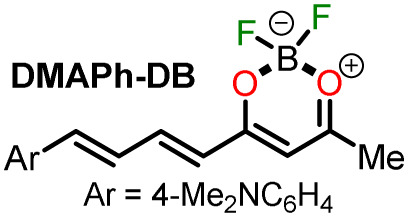	“Turn on”	Gao *et al.*^40^
	2.23 μM, H_2_O	
	*λ*_em_ = 620 nm	
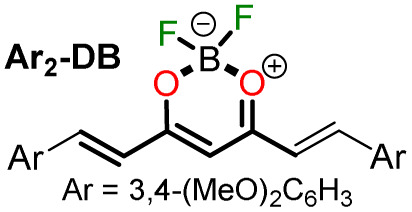	“Turn on”	Chaicham *et al.*^41^
	0.14 μM, H_2_O/MeCN (1 : 4)	
	*λ*_abs_ = 649 nm	
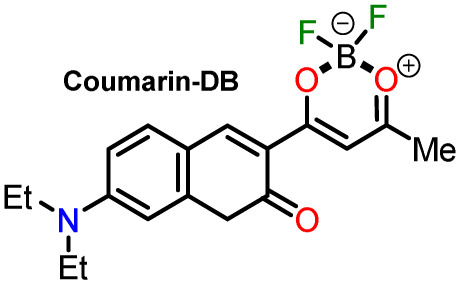	“Turn off”	Li *et al.*^42^
	72 nM, PBS/DMSO (3 : 2, pH 7.4)	
	*λ*_em_ = 565 nm	
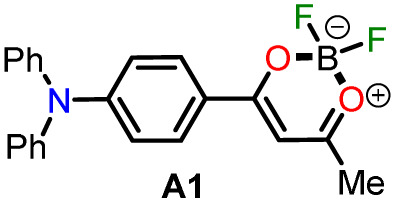	“Turn on”	Tamilarasan *et al.*^19^
	0.36 μM, MeCN/H_2_O (98 : 2)	
	*λ*_em_ = 402 nm	
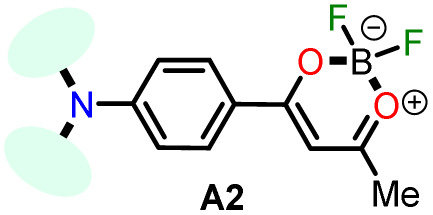	“Turn on–off–on”	This work
	0.18 μM, MeCN/Tris (9 : 1, pH 7.5)	
	*λ*_em_ = 572 nm	

a Data were recorded at different concentrations of A1 and A2 in THF at 20 °C (see eqn (3)).

**Scheme 3:**
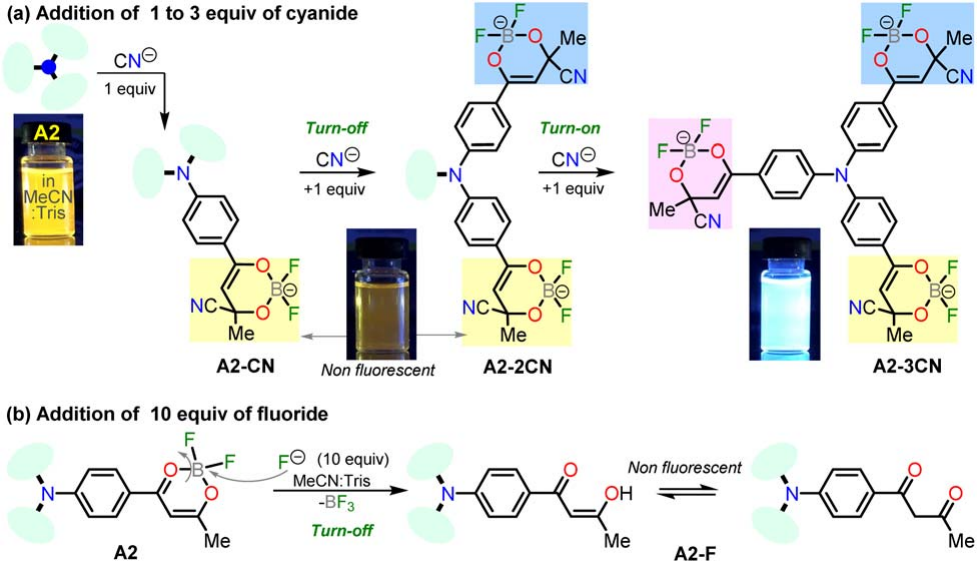
Plausible mechanisms for anions sensing upon addition of (a) 1 to 3 equiv. of CN^−^ or (b) 10 equiv. of F^−^ to solutions of A2.

Notably, the new hybrid dye A2 reported here demonstrates good sensitivity and selectivity with a relatively simple chemical structure and synthetic pathway. Likewise, and due to its trimeric molecular architecture, A2 is one of the few probes that can detect cyanide ions sequentially through a “turn on–off–on” process (*i.e.*, detection by adding 1 equiv. and then completing up to 3 equiv.). Ultimately, the synthesis of A2 is carried out by an operationally simple and efficient process compared to other probes synthesized for cyanide recognition (Schemes 2 and 3).

#### Proposed mechanism for anions recognition

The cyanide sensing mechanism for the dipolar dioxaborinine receptor A1 has already been described as a nucleophilic addition of CN^−^ to the sterically less hindered electrophilic carbon (Scheme 1a).^19^ Upon adding 1 or 2 equiv. of CN^−^ ions to the solution of A2, the probe emission band at ∼570 nm disappears with a turn-off fluorescence due to the symmetry broken into the formed complexes A2-1CN and A2-2CN. However, by adding 3.3 equiv. of CN^−^, the donor–acceptor architecture in A2 disappears, and the symmetry is restored; thus, the emission properties now rely on the less π-conjugated tris-vinyl-TPAm moiety in A2-3CN (*λ*_em_ = 410 nm, Fig. S16†), favoring a redshifted turn-on fluorescence concerning A2 (Scheme 3a). On the other hand, as reported by Yan *et al.*,^43^ the F^−^ addition is presumed to proceed with the opening of the dioxaborinine ring in A2 due to the attack on the boron atom in the probe molecule (Scheme 3b). The mechanistic assumptions cited were tracked by mass spectrometry analysis for adducts A2-1CN, A2-2CN, and A2-F (Fig. S10 and S11†).

#### Test strips

**Fig. 5:**
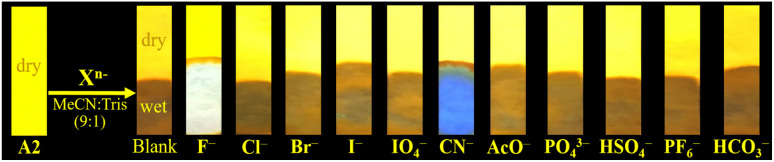
Images of A2 test strips prepared on filter paper for the selective detection of CN^−^ and F^−^ in MeCN/Tris (9 : 1) using a hand-held UV lamp (*λ*_ex_ = 365 nm).

Based on the distinct emission color change of A2 under a 365 nm hand-held UV lamp in an acetonitrile solution by adding cyanide, filter paper immersed with A2 was proposed to detect this anion. The test strip was prepared by simple immersion of qualitative filter paper (Filter Disc, Ref. 3.303.125, Boeco) in a solution of A2 in THF (10 mL at 1.2 mM) followed by air-drying under atmospheric conditions (Fig. 5). Subsequent, anions spiked acetonitrile sample (0.1 mM) was dropped onto the strips for naked eye detection under illumination with a hand-held UV lamp without the paper being completely dry. As depicted, the paper containing A2 displayed bright blue emission only under exposure to cyanide solution. However, the color changed to white after the test strips stained with A2 were immersed into the acetonitrile solutions with fluorine F^−^ (100.0 μM). Based on the distinctive color change of A2 when exposed to CN^−^ this compound proved that the test strips could be applied to detect CN^−^ and F^−^ anions qualitatively in a rapid way.

#### Preliminary two-photon absorption properties

**Fig. 6:**
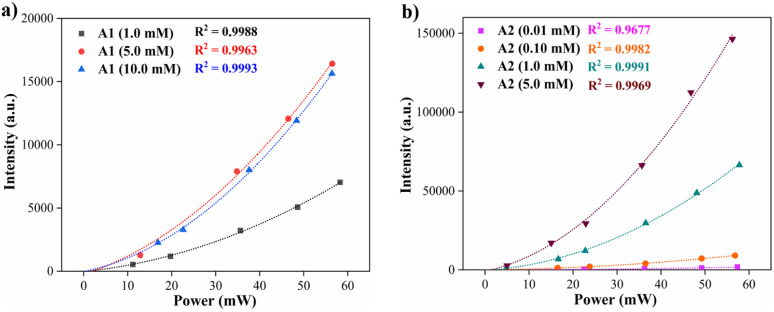
Two-photon induce fluorescence signal as a function of the laser power of compounds (a) A1 and (b) A2 at different concentrations in THF (20 °C).

The preliminary TPA properties of A1 and A2 in THF were studied by detecting two-photon-induced emissions. The detected fluorescence is shown as a function of the laser light power for different concentrations of dyes (Fig. 6). The dots correspond to experimental measurements, and the dashed lines are quadratic fits to the data (*R*^2^/conc. in mM: A1 = 0.999/1.0, 0.996/5.0, and 0.999/10.0; A2 = 0.968/1.0, 0.9982/0.1, 0.999/1.0, and 0.997/5.0). From the fitting parameters, the experimental value for the two-photon excitation action cross-section (*σ*′) can be obtained according to eqn (3) (see the Experimental section). The values obtained for *σ*′ for diverse concentrations of A1 and A2 are also reported (Table 3). The technique was verified by measuring the *σ*′ using rhodamine B (RB) in methanol to 0.05 mM.^44^ The value obtained in the experiment is *σ*′ = 4.4 ± 0.7 GM. Considering the value of *ϕ* reported in the literature for RB,^45^ herein we report a TPA cross-section *δ* = 6.8 ± 1.8 GM that agrees with the previously reported values (Table S2†). Notably, values found for A2 are in the same order of magnitude as RB under similar conditions.

**Table 3:** Two-photon excitation action cross section for dyes A1 and A2^a^

Concentration (mM)	*σ*′ (GM)	
	A1	A2
0.01	—	20 ± 4
0.10	—	12 ± 2
1.00	0.49 ± 0.09	8.8 ± 1.6
5.00	0.25 ± 0.05	4.3 ± 0.8
10.00	0.12 ± 0.02	—

a Data were recorded at different concentrations of A1 and A2 in THF at 20 °C (see eqn (3)).

The results of two-photon absorption experiments demonstrate that the hybrid fluorophores A1 and A2 can induce such processes. The values obtained for the two-photon excitation action cross-section show that trimeric compound A2 has a higher probability of a two-photon induced fluorescence process when compared with the analog monomeric A1. This effect can be attributed to the geometry differences between A1 (dipolar) and A2 (octupolar) since the TPA cross-section in octupoles could scale three times the corresponding values in the isolated dipolar analogs.^17,46–48^ Values of the cross sections in Table 3 change due to variation in the fluorescence quantum yield with the concentration^46^ and the possibility of aggregation effects at higher concentrations. Finally, the respective representative diagrams of the z-scan system and the fluorescence process were made (Fig. S18†) to clarify the optical route by which the fluorescence induced by two-photon occurs. Specifically, Jablonski diagrams in Fig. S18b† show the difference between linear fluorescence and two-photon excitation, clarifying the two-photon absorption mechanism of the fluorophore A2.

## Conclusions

In summary, a highly fluorescent trimeric probe A2 was synthesized using a one-pot methodology, and its photophysical properties were examined. This compound displays high fluorescence quantum yields in solvents with low to medium polarity (*φ* of 0.91 MePh to 0.85 CHCl_3_), interesting emission quantum yield at solid-state (*φ* = 0.24 to 571 nm), and moderate solvatofluorochromism from toluene (485 nm) to acetonitrile (572 nm). The nucleophilic addition reaction of CN^−^ or F^−^ on the dioxaborinine ring changes the emission properties of the A2 solution, giving selective fluorometric detection of these anions with limits of detection of 0.18 and 0.70 μM, respectively. In addition, a test strip assay using A2 has also been applied to detect CN^−^ or F^−^ in organic solutions. Noticeably, CN^−^ can be discriminated from F^−^ by tracking the emission at 410 nm. Ultimately, a clear two-photon induce process was observed for A1 and A2. In particular, the two-photon excitation action cross section of A2 shows that this probe can be exploited, as rhodamine B, in different applications, *e.g.*, two-photon microscopy or non-linear optics for the ion sensing field. The extension of the π-conjugation and the possibility of a symmetry-broken dipolar state induce better photophysical properties in compound A2 than its dipolar analog A1.

## Experimental section

### Reagents and materials

#### Synthesis

Reagents were purchased from commercial sources and used without further purification; these were weighed and handled in the air at room temperature. The reaction was monitored by thin-layer chromatography (TLC), visualized by a UV lamp (254 or 365 nm), and flash chromatography was performed on silica gel (230–400 mesh). The methyl ketone precursor (4-acetylphenyl)diphenylamine (2) was obtained in a 67% yield using a known method starting from triphenylamine (1).^33^ Subsequently, the β-diketone intermediate 1-(4-(diphenylamino)phenyl)butane-1,3-dione (3) was prepared in a 78% yield from methyl ketone 1 (Scheme 2a).^19^

#### Characterization

For the structural characterization of the hybrid compounds A1 and A2, their NMR spectra (Fig. S1–S7†) were recorded at 400 MHz (^1^H), 101 MHz (^13^C{^1^H}), and 374 MHz (^19^F) at 25 °C using CDCl_3_ or DMSO-*d*_6_ as solvents and tetramethylsilane (TMS, *δ*: 0 ppm) as the internal reference. The chemical shifts (*δ*) are reported in ppm, and the coupling constants (*J*) are reported in Hz. The following abbreviations are used for multiplicities: s = singlet, d = doublet, and m = multiplet. The melting point was collected using a capillary melting point apparatus and is uncorrected. The high-resolution mass spectra (HRMS) for the hybrid dyes A1 and A2 (Fig. S8 and S9†) were obtained on an Agilent Technologies Q-TOF 6520 spectrometer *via* electrospray ionization (ESI). The mass spectra for adducts A2-1CN, A2-2CN, and A2-F (Fig. S10 and S11†) were recorded on a Thermo-Scientific LCQ Fleet™ ion-trap mass spectrometer using positive ion mode ESI and a direct inlet system.

Regarding the photophysical studies of A1 and A2, absorption (UV-vis) and emission spectra were recorded at room temperature (20 °C) in an air-equilibrated solution on Varian Cary 100 and Cary Eclipse spectrophotometers, respectively (both are Agilent Technologies devices) using quartz cuvettes with a path length of 1 cm. For fluorescence studies, both the excitation and emission slit widths were 5 nm. The fluorescence quantum yields (*ϕ*) were determined using Prodan^49^ as a reference standard by eqn (1)1
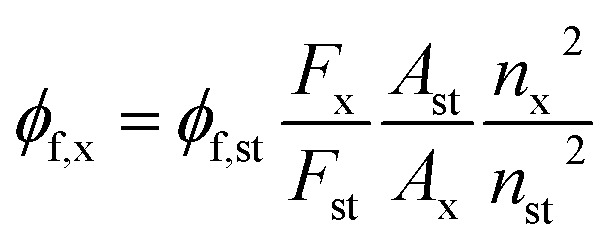
where *F* is the integral photon flux, *A* is the absorption factor, *n* is the solvent refractive index, *ϕ*_f_ is the quantum yield. The indexes x and st denote the sample and standard, respectively.^50^

On the other hand, Catalán's multiparametric relationship can be formulated by eqn (2)

2 *A* = *A*_0_ + *b*SA + *c*SB + *d*SP + *e*SdP

where *A* is a solvent-dependent physicochemical property in a given solvent, and *A*_0_ is the statistical quantity agreeing to the value of the property in the gas phase; SA, SB, SP, and SdP represent independent yet complementary solvent parameters accounting for various types of solute–solvent interactions; and *b* to *e* are the regression coefficients relating the sensitivity of property *A* to the different solute–solvent interaction mechanisms.^51^

Finally, the two-photon excitation action cross section (*σ*′) was measured using a continuous-wave (CW) laser at 817 nm when driving the two-photon transition. The laser light was focused on the sample contained in a 1 mm thick quartz cuvette, employing an objective microscope lens. The sample was placed on a translational stage that allows for implementing the z-scan technique. The fluorescence was detected as a function of the position of the sample by a photomultiplier tube. The dependence of the fluorescence signal (*F*) with the power of the laser light (*P*) is given by eqn (3)3
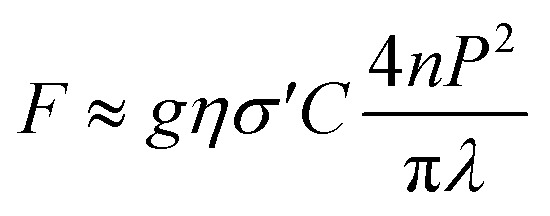
where *g* is the temporal second-order correlation function of the incoming light source, *η* is overall fluorescence collection efficiency, *n* is the refractive index of the solvent, *C* is the sample concentration, and *σ*′ = *ϕδ* (*ϕ* is the quantum yield and *δ* the TPA cross-section).^45^

### Synthesis and characterization

#### 4-(2,2-Difluoro-6-methyl-2*H*-1λ^3^,3,2λ^4^-dioxaborinin-4-yl)-*N*,*N*-diphenylaniline (A1)

To a stirred solution of the freshly synthesized (54% for the two steps from 1) β-diketone 3 (0.12 g, 0.36 mmol) in dichloromethane (DCM, 5.0 mL), BF_3_·OEt_2_ (0.054 mL, 0.44 mmol) was added dropwise at room temperature and maintained for 30 min. The reaction mixture color changed instantly to red during the BF_3_·OEt_2_ addition. The reaction was quenched with aqueous NaOH solution 0.5 M and extracted with DCM × 3. The crude product was purified by column chromatography in silica gel (DCM as eluent) to obtain A1 as a red solid in a 96% yield. Mp: 209–212 °C. ^1^H NMR (401 MHz, CDCl_3_): *δ* = 2.33 (s, 3H), 6.37 (s, 1H), 6.94 (d, *J* = 8.9 Hz, 2H), 7.26–7.17 (m, 6H), 7.38 (t, *J* = 7.7 Hz, 4H), 7.94–7.80 (m, 2H) ppm. ^13^C NMR (100 MHz, CDCl_3_): *δ* = 24.4, 95.9, 118.3, 121.5, 126.0, 126.7, 130.0, 131.3, 145.3, 154.5, 180.9, 188.2 ppm. ^19^F NMR (374 MHz, CDCl_3_): *δ* = −139.9 ppm. HRMS (ESI) *m*/*z*: [M − F]^+^ calcd for C_22_H_18_BFNO_2_^+^ 358.1415; found 358.1409. These data matched those previously reported.^19^

#### Tris(4-(2,2-difluoro-6-methyl-2*H*-1λ^3^,3,2λ^4^-dioxaborinin-4-yl)phenyl)amine (A2)

To a stirred solution of triphenylamine (1, 0.245 g, 1.0 mmol) in acetic anhydride (1.0 mL), BF_3_·OEt_2_ (0.5 mL) was added dropwise at 60 °C and maintained for 5 h. The reaction mixture color changed instantly to black during the addition of BF_3_·OEt_2_. The reaction was quenched with aqueous NH_3_ solution 0.5 M and extracted with DCM × 3. The crude product was purified by column chromatography in silica gel (DCM as eluent) to obtain A2 as an orange solid in a 52% yield. Mp: 210–211 °C. ^1^H NMR (400 MHz, DMSO-*d*_6_) *δ*: 2.45 (s, 9H), 7.23 (s, 3H), 7.36 (d, *J* = 8.5 Hz, 6H), 8.20 (d, *J* = 8.5 Hz, 6H) ppm. ^13^C NMR (101 MHz, DMSO-*d*_6_) *δ*: 24.4 (CH_3_), 97.9 (CH), 124.9 (CH), 126.4 (C), 131.3 (CH), 151.4 (C), 179.7 (C), 193.2 (C=O) ppm. ^19^F NMR (374 MHz, CDCl_3_): *δ* = −137.8 ppm. HRMS (ESI) *m*/*z*: [M − F]^+^ calcd for C_30_H_24_B_3_F_5_NO_6_^+^ 622.1798; found 622.1816.

## Conflicts of interest

The authors declare no competing financial interest.

## Supplementary Material

RA-013-D2RA07498B-s001
