# Supplementary material for: Photophysical and anion sensing properties of a triphenylamine–dioxaborinine trimeric compound
Source: RSC Adv. 2023 Jan 9;13(3):1757–64. doi: 10.1039/d2ra07498b (PMC9828043; doi:10.1039/d2ra07498b)
Supplement: RA-013-D2RA07498B-s001 [file RA-013-D2RA07498B-s001.pdf]

Electronic Supplementary Material (ESI) for RSC Advances.
This journal is © The Royal Society of Chemistry 2022

Electronic supplementary information (ESI)

Photophysical and anions sensing properties of a triphenylamine–dioxaborinine trimeric compound

Alexis Tigreros,[a] Camilo Bedoya-Malagón,[b] Alejandra Valencia,[b] Mayerlin Núñez-Portela,[b] and Jaime Portilla*[a]

[a] Bioorganic Compounds Research Group, Department of Chemistry, Universidad de Los Andes, Carrera 1 No. 18A-10, Bogotá 111711, Colombia

[b] Quantum Optics Laboratory, Department of Physics, Universidad de los Andes, Carrera 1 No. 18A-10, Bogotá, Colombia

*E-mail: jportill@uniandes.edu.co; Tel: +57 1 3394949. Ext. 2080

Contents

1. Copies of NMR spectra 2

2. HRMS and MS analysis 8

3. Photophysical properties of compounds A1 and A2 9

3.1. Catalán's multiparametric scale 9

3.2. Cyanide sensing properties of compound A2 10

3.3. Two-photon absorption measurements 12

References 13

1. Copies of NMR spectra

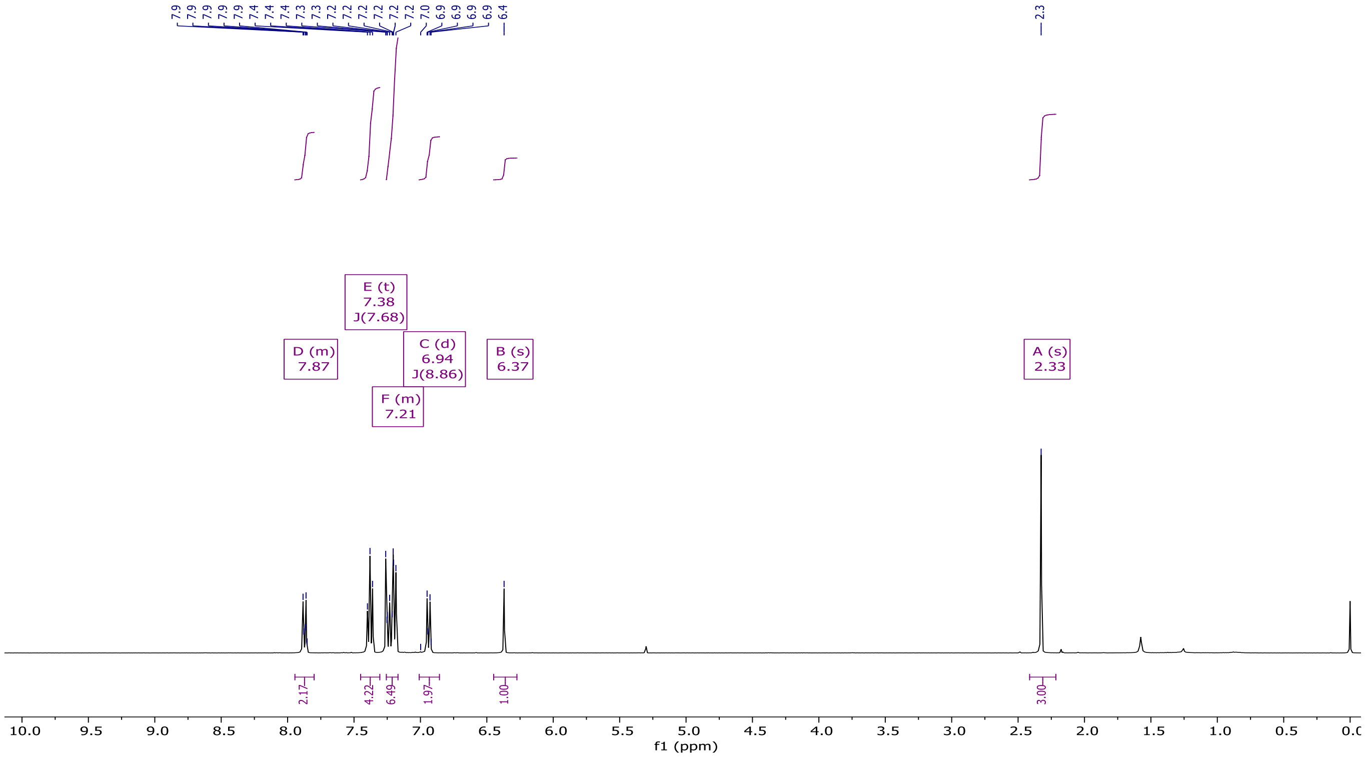

Fig. S1 ^{1}H NMR spectrum for compound **A1** in $CDCl_3$ (400 MHz).

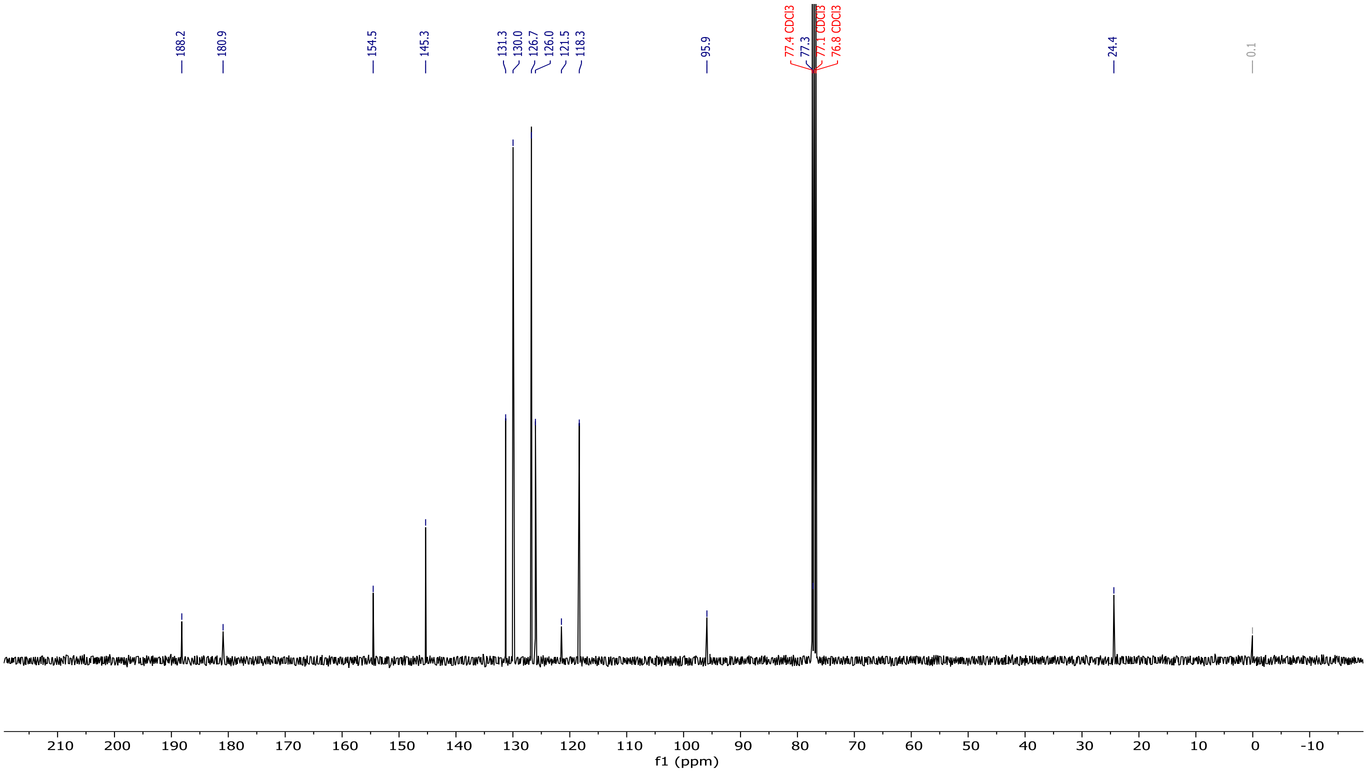

Fig. S2 ^{13}C NMR spectrum for compound **A1** in $CDCl_3$ (100 MHz).

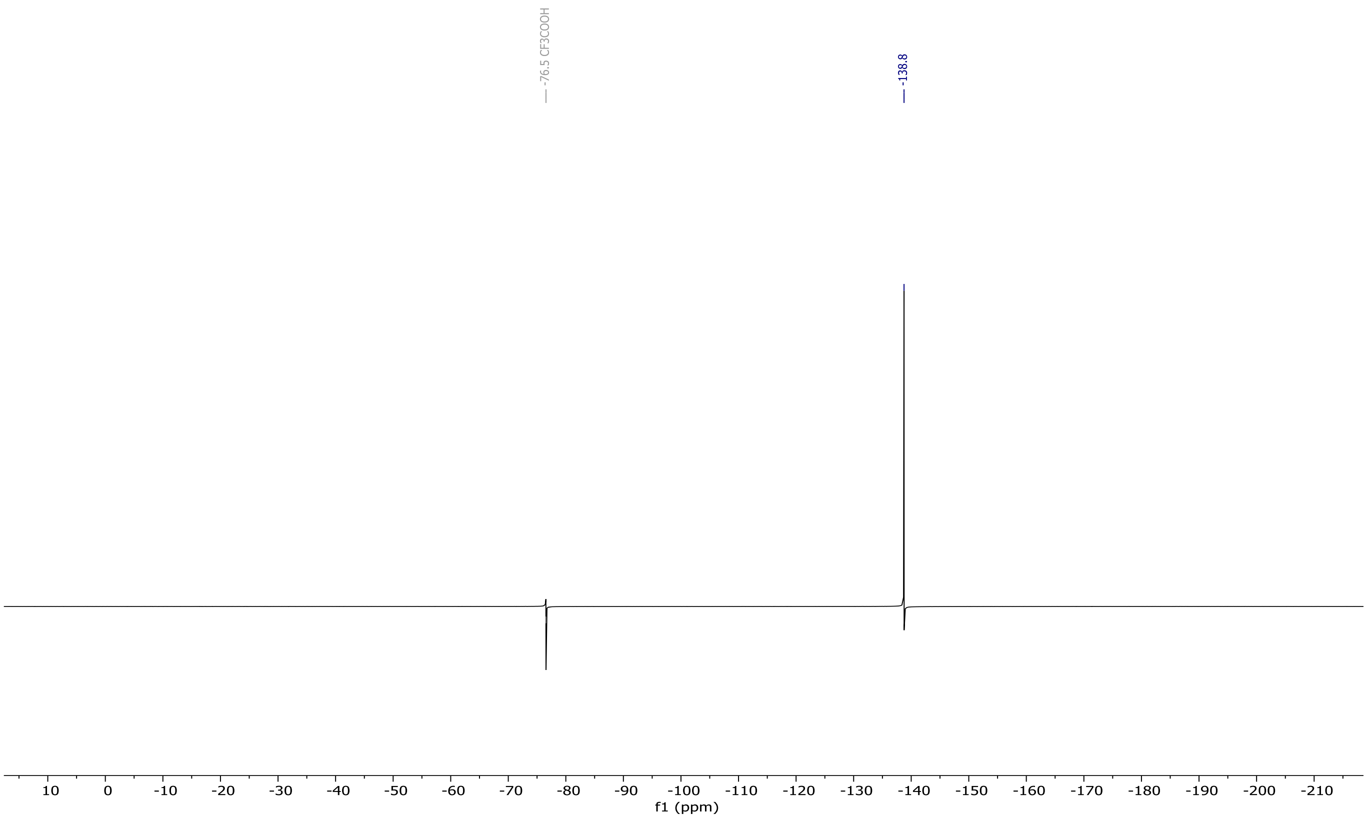

Fig. S3 ^{19}F NMR spectrum for **A1** in $CDCl_3$ (374 MHz) using TFA as internal standard.

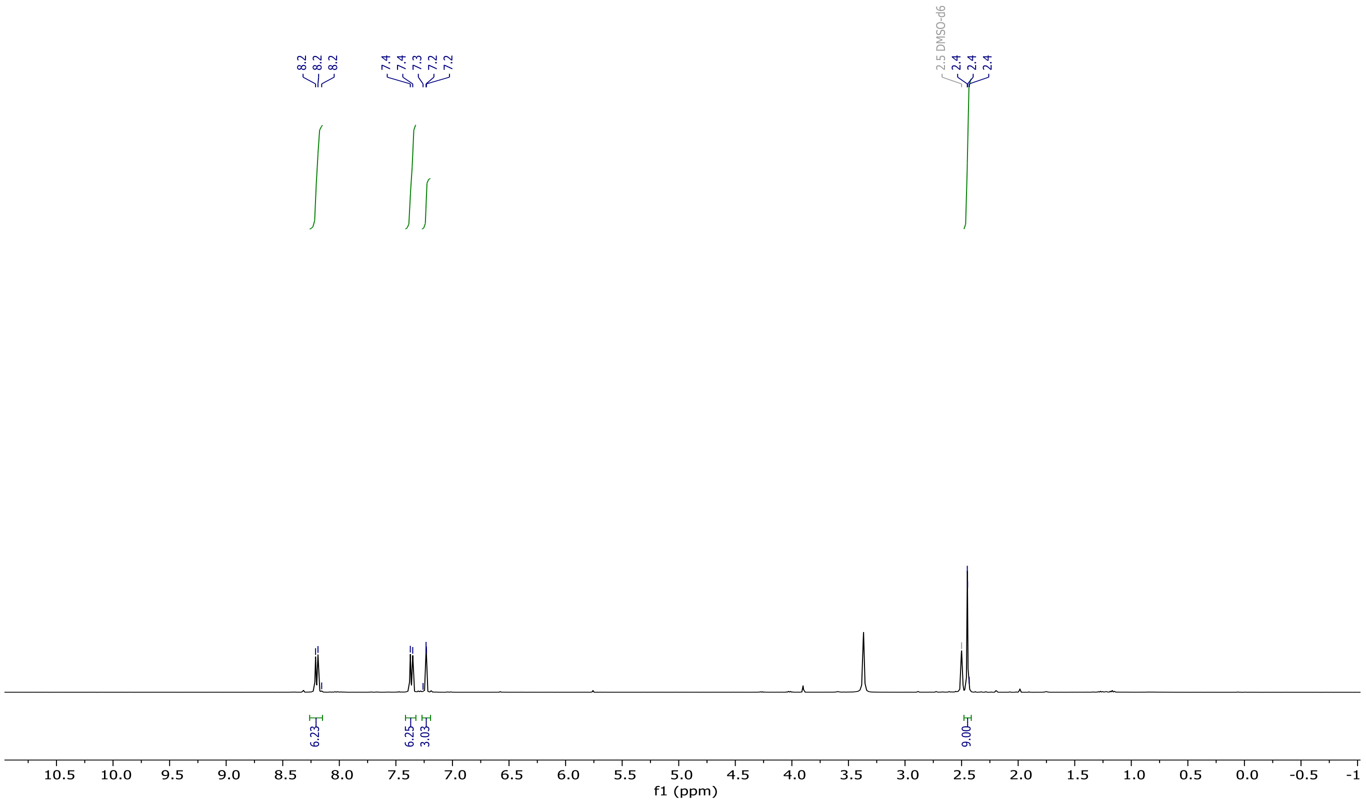

Fig. S4 ^{1}H NMR spectrum for compound **A2** in DMSO-d_6 (400 MHz).

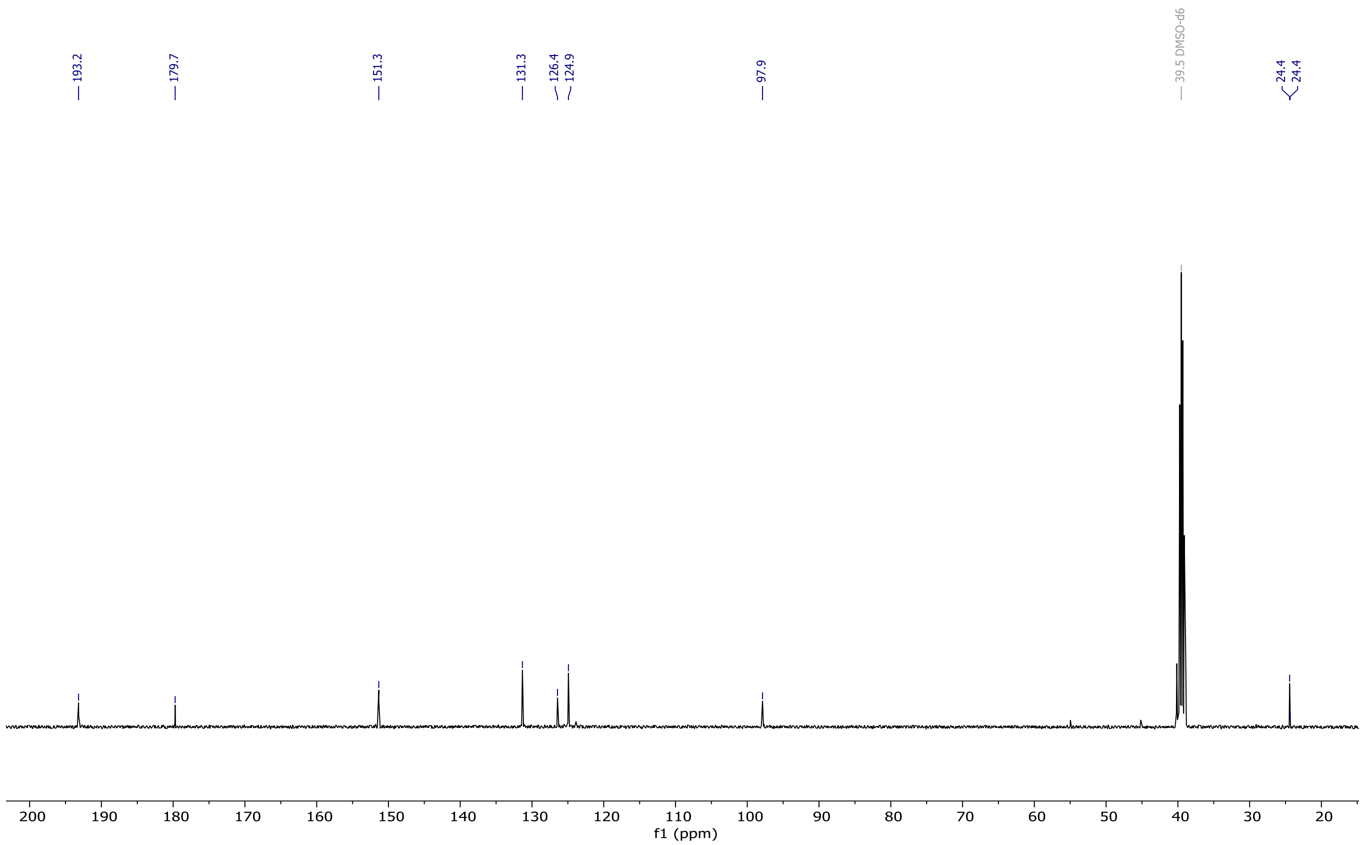

Fig. S5 ^{13}C NMR spectrum for compound **A2** in DMSO-d_6 (100 MHz).

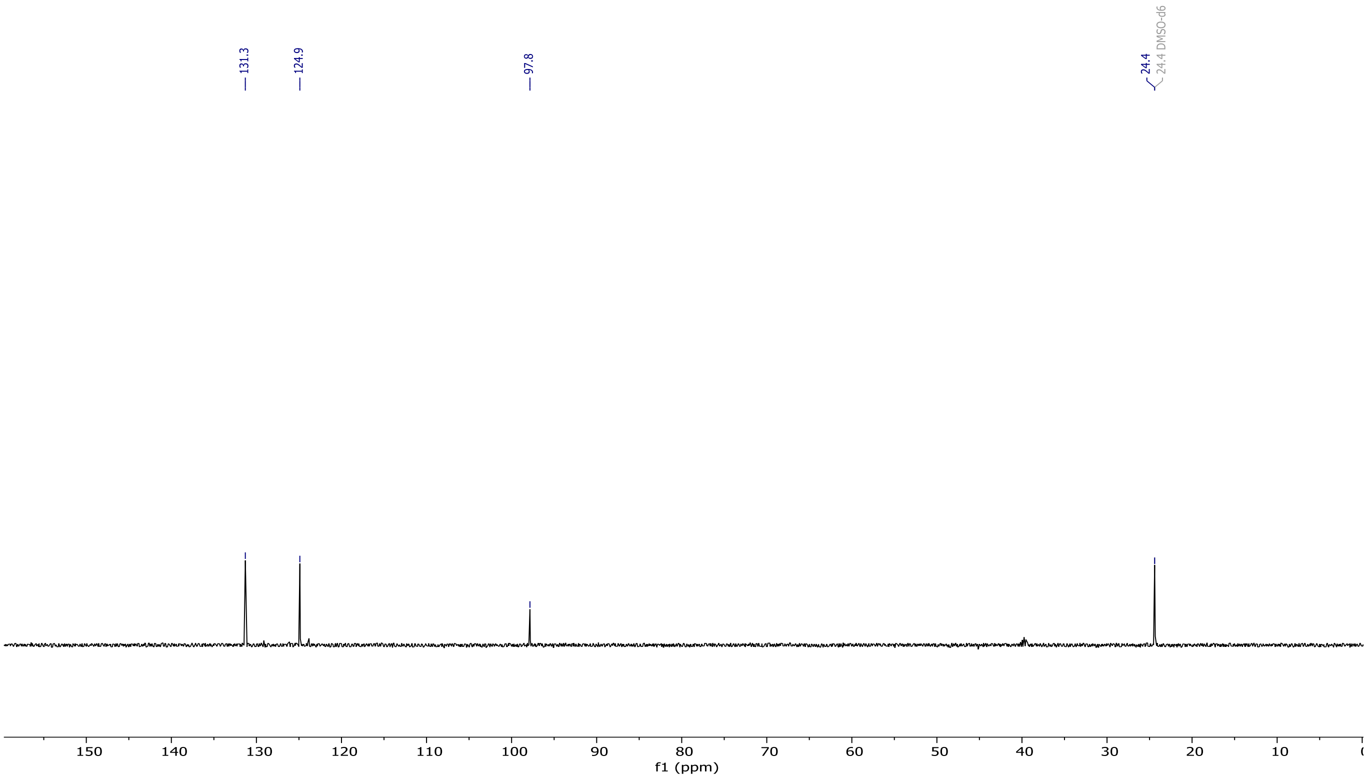

Fig. S6 DEPT-135 spectrum for compound **A2** in DMSO-d_6 (100 MHz).

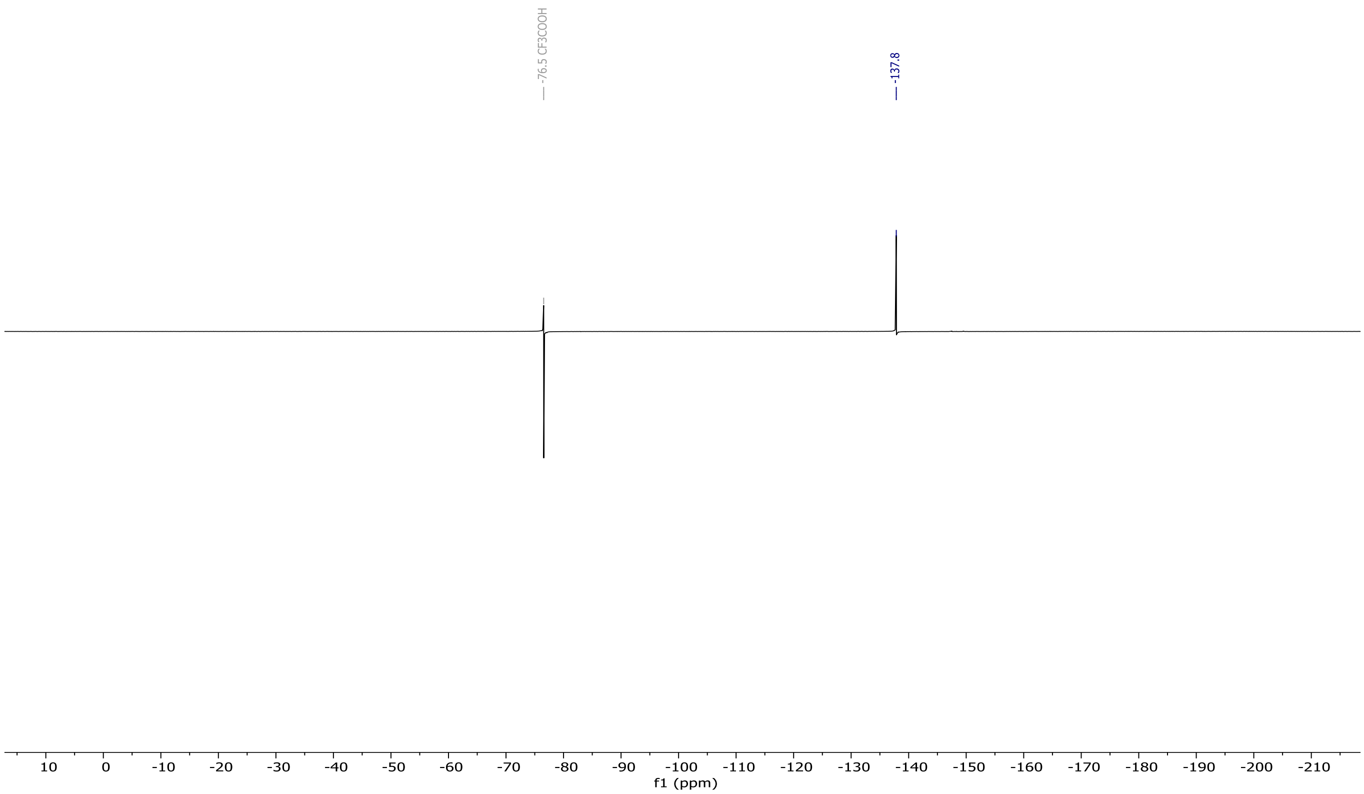

Fig. S7. ^{19}F NMR spectrum for **A2** in DMSO-d_6 (374 MHz) using TFA as an internal standard.

2. HRMS and MS analysis

**Data Filename**	ATO-6.d	**Sample Name**	ATO-6
**Sample Type**	Sample	**Position**	P1-F6
**Instrument Name**	Instrument 1	**User Name**	
**Acq Method**	Default 2021.m	**Acquired Time**	7/1/2022 1:55:27 PM
**IRM Calibration Status**	Success	**DA Method**	Metodo-analisis-signaltonoise.m
**Comment**			

Sample Group **Info.**

User Chromatograms

Fragmentor Voltage 175 **Collision Energy** 0 **Ionization Mode** ESI

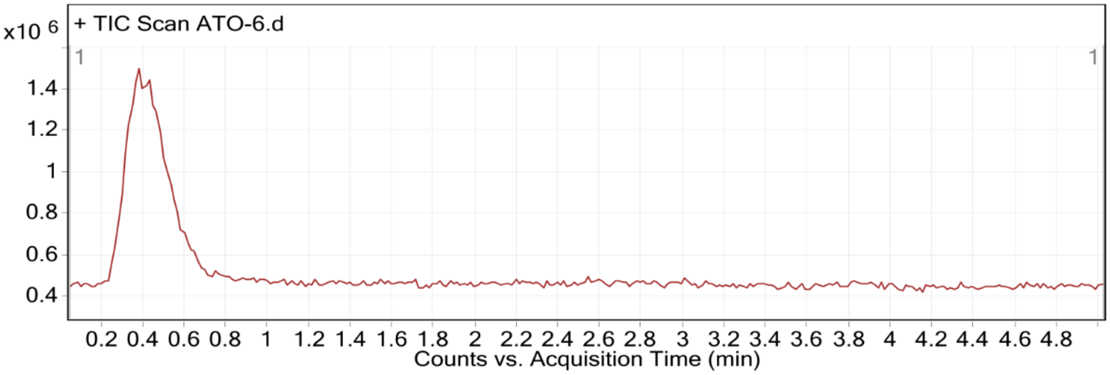

User Spectra

Fragmentor Voltage 175 **Collision Energy** 0 **Ionization Mode** ESI

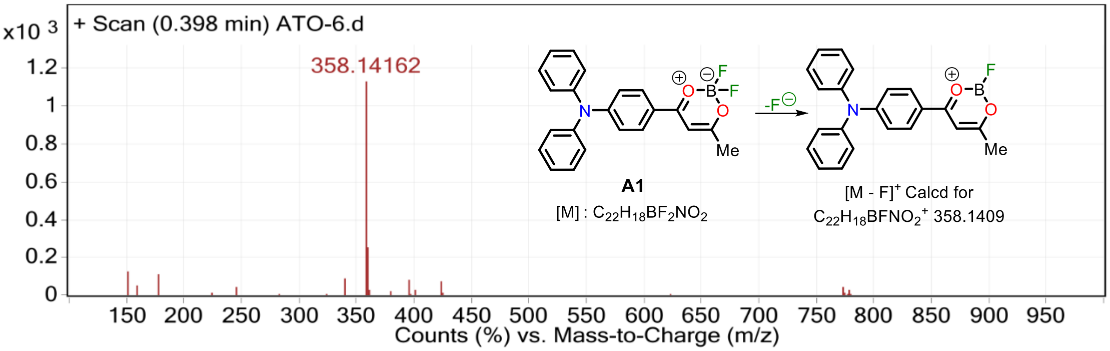

Peak List

m/z	z	Abund
150.02652		37686.3
158.15358		16670.3
177.05479		32164.6
338.3415		27140.6
357.14381		70412.1
358.14162	1	309354.9
358.29809		16875.6
359.14394	1	72178.1

Fig. S8 HRMS analysis for compound **A1**.

**Data Filename**	ATO-7 2.d	**Sample Name**	ATO-7
**Sample Type**	Sample	**Position**	P1-F7
**Instrument Name**	Instrument 1	**User Name**	
**Acq Method**	Default 2021.m	**Acquired Time**	7/1/2022 5:06:53 PM
**IRM Calibration Status**	Success	**DA Method**	Metodo-analisis-signaltonoise.m
**Comment**			

Sample Group **Info.**

User Chromatograms

Fragmentor Voltage 175 **Collision Energy** 0 **Ionization Mode** ESI

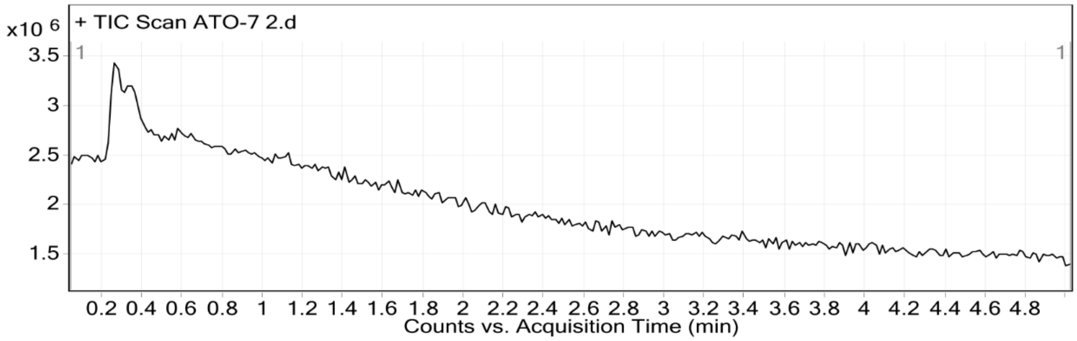

User Spectra

**Fragmentor Voltage**	**Collision Energy**	**Ionization Mode**
175	0	ESI

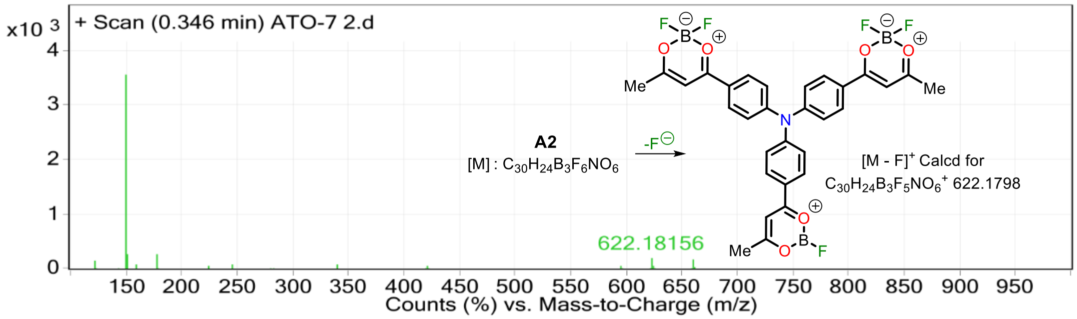

Peak List

m/z	z	Abund
121.02966		48074.2
149.02399	1	951837.1
149.05495		51891.7
150.02696	1	76982.4
177.05474		76592.2
622.18156		59360.9
659.21687		54362

Fig. S9 HRMS analysis for compound **A2**.

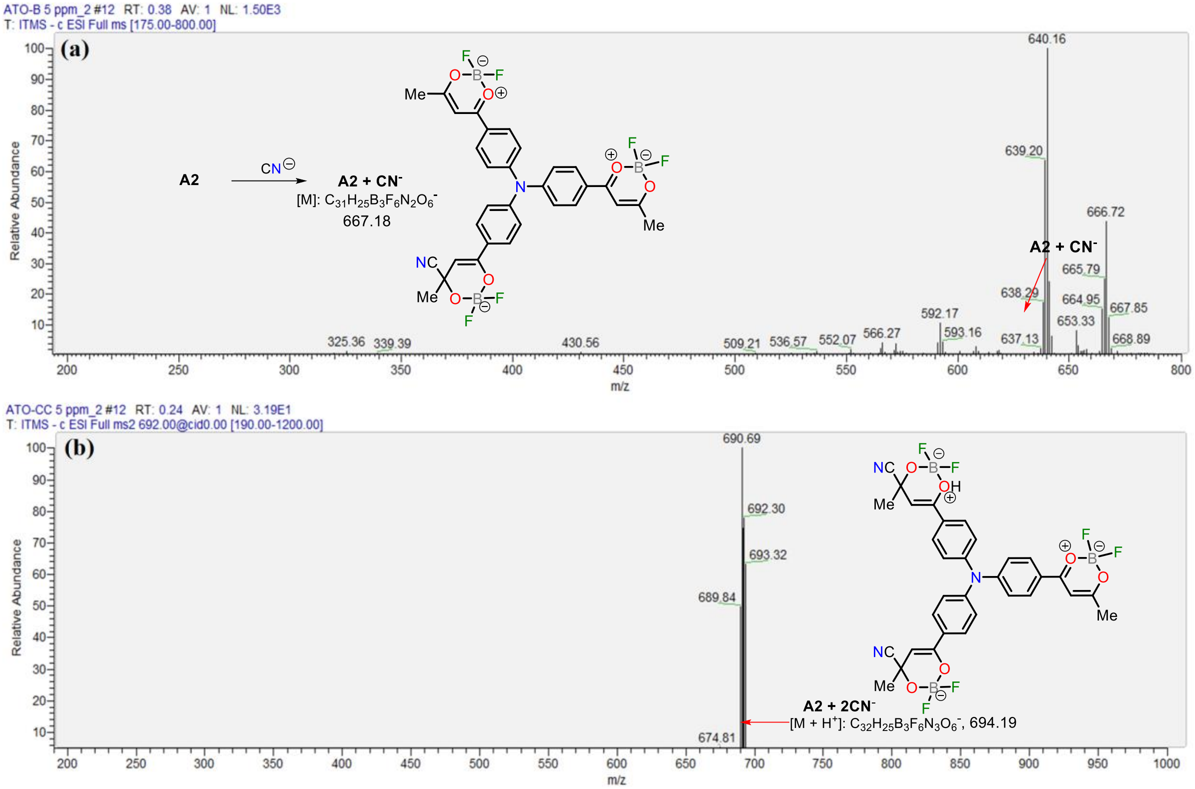

Fig. S10 MS analysis for **A2** upon addition of a) 0.5 equiv and b) 2.0 equiv of $Bu_4N^+CN^-$ in MeCN.

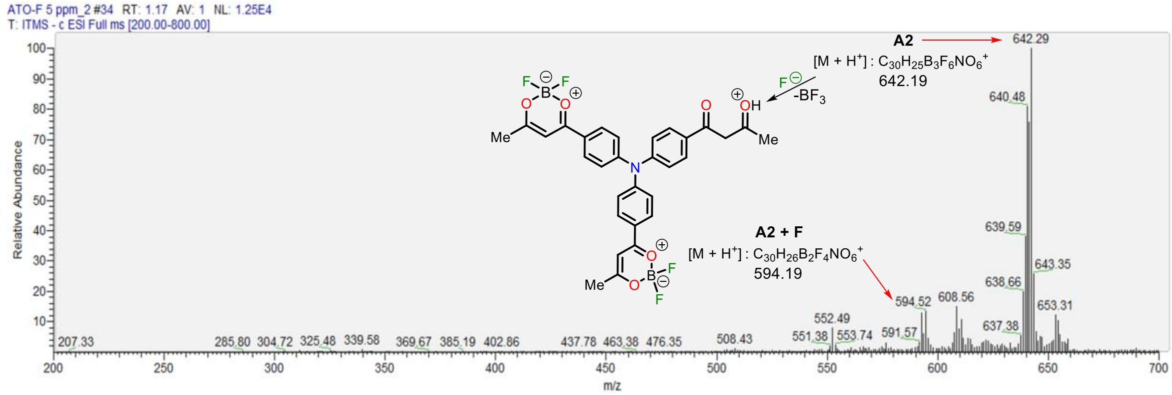

Fig. S11 MS analysis for **A2** upon addition of 1.0 equiv of $Bu_4N^+F^-$ in MeCN. Ion-trap using positive ion mode.

3. Photophysical properties of compounds A1 and A2

3.1. Catalán's multiparametric scale

Table S1. Catalán solvent parameters and emission maximum wavelengths of **A1** and **A2**.[a]

Solvent	SP	SdP	SA	SB	A1, Emax (cm^{-1})	A2, Emax (cm^{-1})
TBME	0.622	0.422	0.000	0.567	21692	20661
MePh	0.782	0.284	0.000	0.128	21413	20325
THF	0.714	0.634	0.000	0.591	21277	19608
EA	0.656	0.603	0.000	0.542	21276	19157
$CHCl_3$	0.614	0.783	0.047	0.071	20833	18832
DMF	0.759	0.977	0.031	0.613	20325	17637
MeCN	0.645	0.974	0.044	0.286	20325	17391

[a] Experiments were carried out in various solvents of different polarities. EA = ethyl acetate, Emax = Emission maximum.

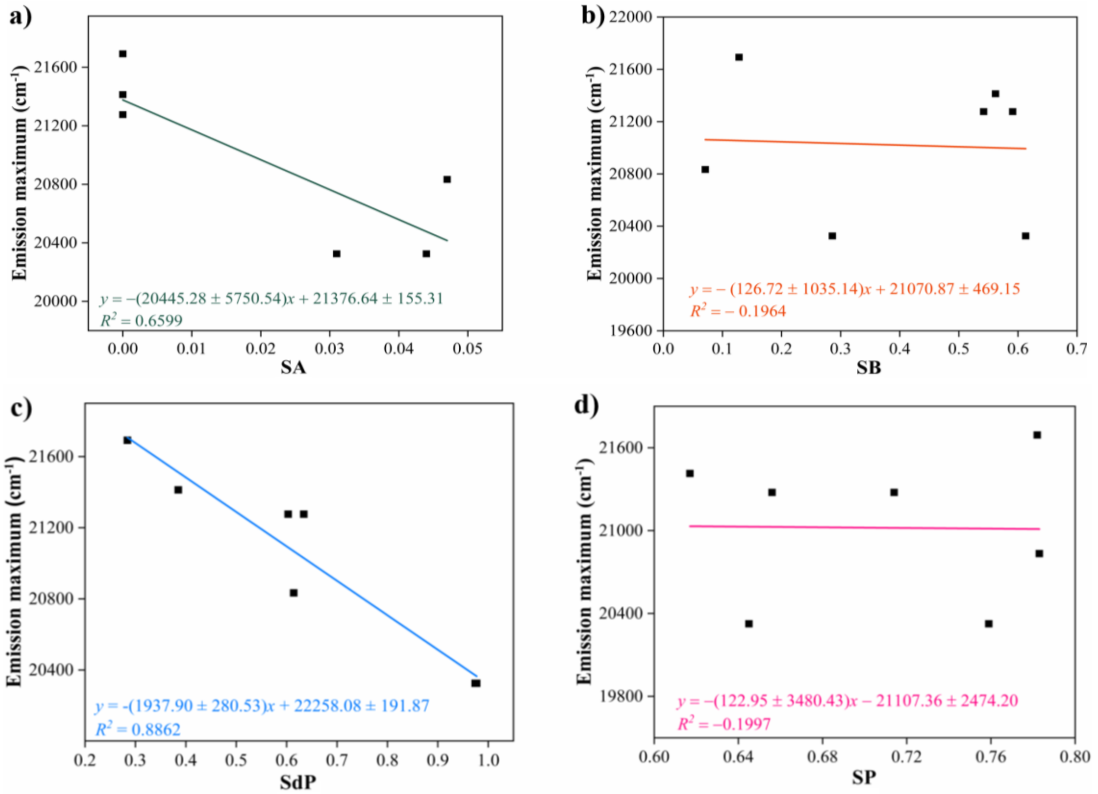

Fig. S12 Plots of emission maximum vs. a) Solvent dipolarity (SdP), b) Solvent polarizability (SP), c) Solvent acidity (SA), and d) Solvent basicity (SB) for compound **A1**.

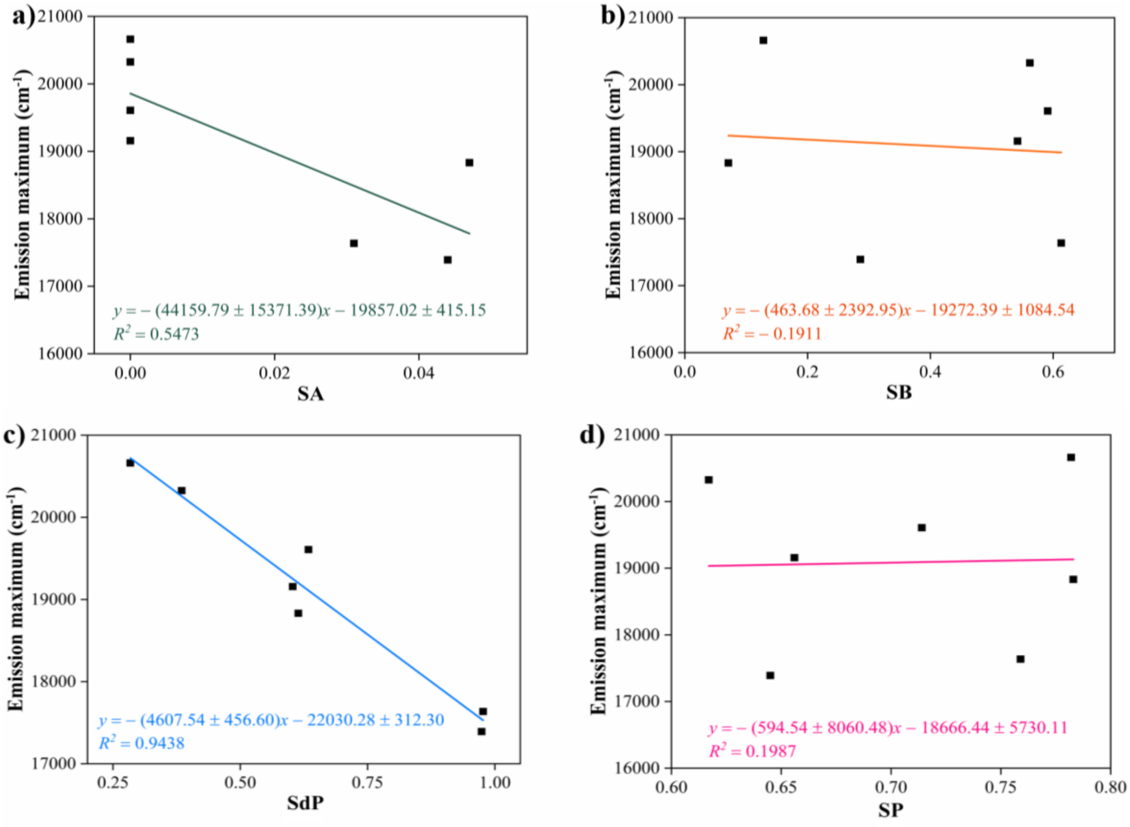

Fig. S13 Plots of emission maximum vs. a) Solvent dipolarity (SdP), b) Solvent polarizability (SP), c) Solvent acidity (SA), and d) Solvent basicity (SB) for compound **A2**.

3.2. Cyanide sensing properties of compound A2

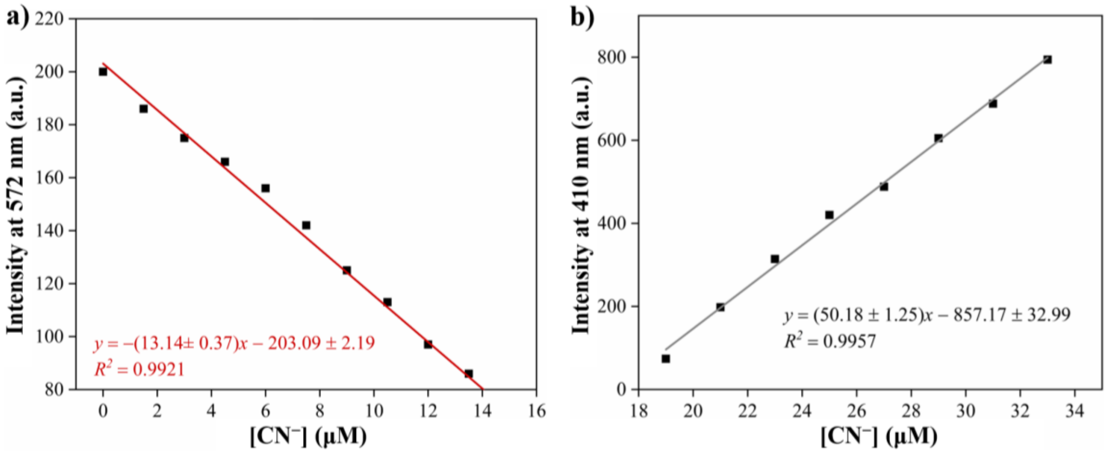

Fig. S14 Linear plot of emission intensities of **A2** (10 μM) in acetonitrile solution in the presence of CN^- a) concentration range of CN^- 0-10 μM and b) 19-33 μM

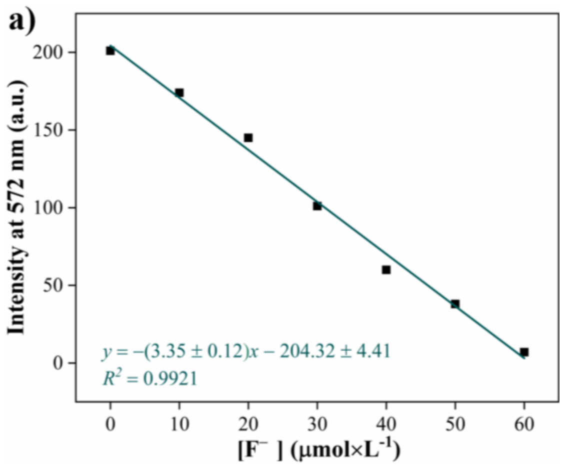

Fig. S15 Linear plot of emission intensity of **A2** (10 μM) in MeCN for F^- (0-60 μM).

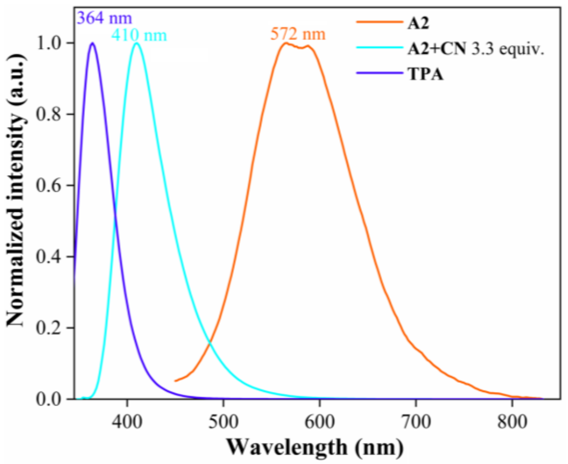

Fig. S16 Normalized emission spectra of **A2**, **A2** + 3.3 equiv of CN and triphenylamine (**TPA**) in MeCN at 20 °C, λ_{ex} = 350 nm for **A2** and **A2** + **CN**, and λ_{ex} = 300 nm for **TPA**.

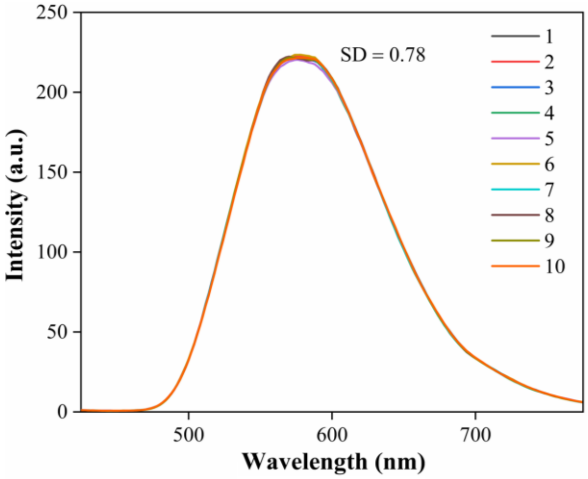

Fig. S17 Ten emission spectra measurements for LOD of CN^- and F^- determination of **A2**.

3.3. Two-photon absorption measurements

To measure the two-photon induce fluorescence in compounds **A1** and **A2**, an external cavity diode laser (ECDL) in Littrow configuration at 817 nm is used to excite the two-photon transition. The light beam was focused on the sample using a microscope objective (Edmund Optics 58-373). Different probe concentrations in THF were contained in a 1 mm thick quartz cuvette. The cuvette was placed on a translation stage allowing small and precise sample movements along the laser light path. The translational stage permits pinpointing the focal point of the laser light inside the sample by performing a z-scan. The emission is detected as a function of the position of the sample by a photomultiplier tube (PMT Hamamatsu R5929).[1] The fluorescence signal maximum is reported as fluorescence in Fig. 6. Spectral filters remove the scatter of infrared light from the excitation laser.

The excitation action cross section is obtained from the fitting parameters in Figure 6a-b and Eq. 3. For laser light $g = 1$, the overall fluorescence collection efficiency is $\eta = 0.0018 \pm 0.0002$ for **A1** and $\eta = 0.0011 \pm 0.0002$ for **A2**, depending on the spectral sensitivity of the PMT, and the refractive index for THF is $n = 1.4072$. The experimental technique was verified by measuring the two-photon absorption cross-section (δ) in Rhodamine B in methanol. Table S2 shows the value of the TPA cross section measured in this work compared to previously reported values. The values show that the technique employed in this work allows for the measurement of two-photon absorption cross sections.

Table S2 Reported values of Two-photon absorption cross sections for Rhodamine in MeOH.

Concentration	λ (nm)	δ (GM)	Reference
0.05 mM	817	6.8±1.8	This work
0.001 mM	798	12.2	[2]
0.0382 mM	802	10.4	[3]

It is important to note that the respective representative diagrams of the z-scan system (Fig. S7a) and the fluorescence process (Fig. S7b) were made to clarify the optical route by which the fluorescence induced by two-photon occurs in the studied compounds.

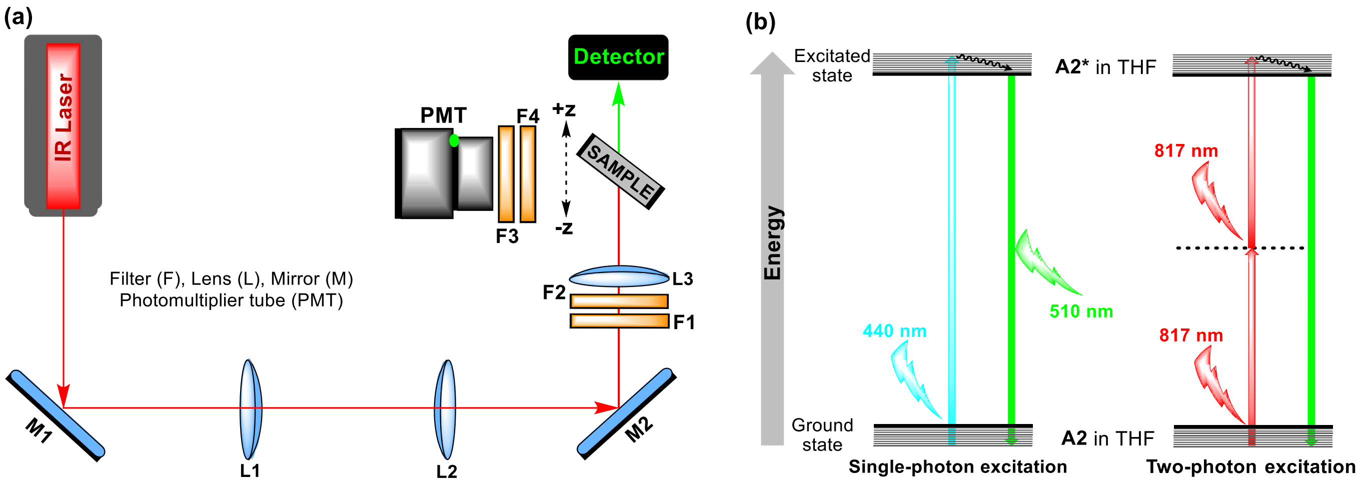

Fig. S18 a) Experimental setup to measure the two-photon absorption excitation action cross section. b) Jablonski diagrams showing the single-photon (left) and two-photon (right) excitations for compound **A2** in THF.

References

1. C. B. Malagon, M. Pellaton, A. Tigreros, J. Portilla, A. Valencia and M. Nunẽz Portela, in *OSA Nonlinear Optics 2021*, ed. R. Boyd Conti, C., Christodoulides, D., and Rakich, P., Optica Publishing Group, Washington, DC, 2021, p. NTh3A.13.
2. C. Xu and W. W. Webb, *Journal of the Optical Society of America B*, 1996, **13**, 481–491.
3. D. A. Oulianov, I. V Tomov, A. S. Dvornikov and P. M. Rentzepis, *Optics Communications*, 2001, **191**, 235–243.
